# Antimicrobial Susceptibility and Distribution Characteristics of *Mycoplasma pneumoniae* Isolates in Beijing, China, from 2017 to 2025

**DOI:** 10.3390/ph19030488

**Published:** 2026-03-16

**Authors:** Chao Yan, Yujie Chen, An Su, Xuanfeng Liu, Xinyu Jia, Xue Ren, Hanqing Zhao, Yanling Feng, Jinghua Cui, Yu Sun, Linqing Zhao, Jing Yuan

**Affiliations:** 1 Department of Bacteriology, Capital Center for Children Health, Capital Medical University, Capital Institute of Pediatrics, Beijing 100020, China; 2 Capital Institute of Pediatrics-Peking University Teaching Hospital, Beijing 100020, China; 3 Capital Institute of Pediatrics, Chinese Academy of Medical Sciences, Peking Union Medical College, Beijing 100020, China; 4 Department of Virology, Capital Center for Children Health, Capital Medical University, Capital Institute of Pediatrics, Beijing 100020, China

**Keywords:** *Mycoplasma pneumoniae*, *Mycoplasma pneumoniae* pneumonia, minimum inhibitory concentrations, macrolide resistance, children

## Abstract

**Background/Objectives**: The aim of this study was to clarify the antimicrobial susceptibility and distribution characteristics of *Mycoplasma pneumoniae* (*M. pneumoniae*, MP) collected from children in Beijing, China, from 2017 to 2025. **Methods**: A total of 197 MP isolates were analyzed. Mutations in macrolide-resistant loci of MP strains were detected via real-time fluorescent quantitative polymerase chain reactions. We used the broth microdilution method to determine the minimum inhibitory concentrations (MICs) of erythromycin, azithromycin, tetracycline, levofloxacin, and moxifloxacin against these isolates. The distribution characteristics of MIC values were further analyzed according to the isolates’ collection year, epidemic phase (low epidemic phase, epidemic initiation phase, ultra-low epidemic phase, outbreak phase, and epidemic recovery phase), and the corresponding patient age group (<3 years, 3–6 years, and ≥6 years). **Results:** All 197 isolates were found to be resistant to erythromycin and azithromycin, with a resistance rate of 100%. In contrast, the strains remained susceptible to tetracycline, levofloxacin and moxifloxacin. The highest resistance rate was 100% for macrolides. The MIC_90_ values were 1024 μg/mL for erythromycin, 256 μg/mL for azithromycin, 0.5 μg/mL for tetracycline, 1 μg/mL for levofloxacin, and 0.125 μg/mL for moxifloxacin, respectively. Distinct differences in MIC distributions of erythromycin and azithromycin were observed across collection years, epidemic phases, and age groups. **Conclusions**: The resistance of MP to macrolides in children is closely associated with the epidemic intensity and age of the patient. Erythromycin is no longer suitable as an empirical therapy for MP infections during epidemic periods, whereas azithromycin can be cautiously administered in young children according to age stratification and MIC detection results. Meanwhile, it is imperative to strengthen the prevention and control of cluster MP infections during epidemic phases to reduce the transmission of drug-resistant MP strains.

## 1. Introduction

*Mycoplasma pneumoniae* (*M. pneumoniae*, MP) is one of the most common etiologic agents of respiratory tract infections in children, with a particularly high incidence among pediatric and adolescent populations [1,2]. MP-associated pneumonia accounts for 10–30% of all cases of childhood community-acquired pneumonia (CAP) [3], and the pathogen may also induce a number of extrapulmonary complications, including myocardial injury, hepatic dysfunction, renal impairment, encephalitis, gastrointestinal dysfunction, and arthritis [4,5]. MP epidemics occur in a cyclical pattern every 3–7 years [6]; from late 2019 to early 2020, outbreaks of MP infections emerged across multiple countries, predominantly in Europe and Asia [7,8]. However, during the coronavirus disease 2019 (COVID-19) pandemic from 2020 to 2022, the implementation of non-pharmaceutical interventions led to a dramatic decline in positive *M. pneumoniae* detection rates across 20 countries in Asia, Oceania, the Americas, and Europe [9,10]. In 2023, nevertheless, widespread *M. pneumoniae* outbreaks were reported worldwide, including in China, Denmark, the United States, and the Netherlands [11,12,13].

Macrolide antibiotics are first-line agents for the treatment of *M. pneumoniae* infections. Owing to their high oral bioavailability and once-daily dosing formulations, these agents have been widely adopted in outpatient clinical practice. Over the past two decades, however, macrolide-resistant *Mycoplasma pneumoniae* (MRMP) has emerged successively worldwide, with the most severe prevalence in Asia. In some epidemic years in Japan and China, the detection rate for MRMP exceeded 90% [14,15]. Currently, the clinical management of MRMP infections poses a major therapeutic challenge. The present study aimed to analyze antimicrobial susceptibility and the distribution characteristics of *M. pneumoniae* collected from children in Beijing, China, from 2017 to 2025 across distinct epidemic periods and pediatric age groups.

## 2. Results

### 2.1. Overall Antimicrobial Susceptibility of MP Isolates, 2017–2025

A total of 197 clinical *M. pneumoniae* isolates were included in this study, with in vitro antimicrobial susceptibility testing performed for all five commonly used antibiotics. Among these isolates, 197 (100%) were resistant to erythromycin and azithromycin. The detection of macrolide resistance-associated mutations in the V region of the 23S rRNA gene revealed the presence of the A2063G mutation in 100% of the isolates. For resistant isolates, the MIC ranges of erythromycin and azithromycin were 4–1024 μg/mL and 1–512 μg/mL, with MIC_50_ values of 512 μg/mL and 64 μg/mL and MIC_90_ values of 1024 μg/mL and 256 μg/mL, respectively (Table 1). The MIC_50_ and MIC_90_ of erythromycin were 8- and 4-fold higher than those of azithromycin, respectively (Table 1). All isolates (100%) were susceptible to tetracycline, levofloxacin, and moxifloxacin, with MIC ranges of ≤0.125–2 μg/mL, ≤0.125–1 μg/mL, and ≤0.125–0.5 μg/mL, respectively (Table 1).

Erythromycin MIC values were divided into three categories: low-level resistance (≤64 μg/mL), moderate-level resistance (128–256 μg/mL), and high-level resistance (512–1024 μg/mL). Azithromycin MIC values were also classified as low-level resistance (≤16 μg/mL), moderate-level resistance (32–128 μg/mL), and high-level resistance (256–512 μg/mL). For erythromycin, 98.5% (194/197) of MRMP isolates had an MIC ≥128 μg/mL, and 70.6% (139/197) had an MIC ≥512 μg/mL. For azithromycin, 69.5% (137/197) of MRMP isolates exhibited an MIC ≥32 μg/mL, and 13.7% (27/197) had an MIC ≥256 μg/mL. These findings indicated that MRMP isolates displayed a high level of in vitro resistance to erythromycin (Table 2).

### 2.2. Comparison of Antimicrobial Susceptibility in Macrolide-Resistant Mycoplasma pneumoniae Isolates Across Different Years

**Figure 1:**
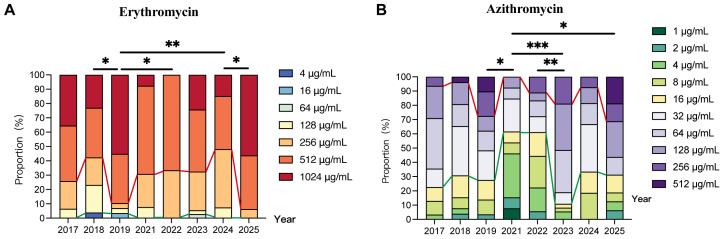
Distribution characteristics of erythromycin and azithromycin MICs in *Mycoplasma pneumoniae* isolates across different years in Beijing, China (2017–2025). Strains below the green line were low-concentration resistant, and those above the red line were high-concentration resistant. (**A**) Erythromycin; (**B**) Azithromycin. * *p* < 0.05, ** *p* < 0.01, *** *p* < 0.001.

In terms of overall antimicrobial susceptibility levels, the MIC_50_ of erythromycin remained ≥512 μg/mL throughout 2017–2025, and the MIC_90_ reached 1024 μg/mL in most years, indicating a state of high-level resistance (Table 3). In contrast, the MIC_50_ and MIC_90_ values of azithromycin were lower; the highest values observed from 2017 to 2025 were 128 μg/mL and 512 μg/mL, respectively (Table 3). The MICs of erythromycin and azithromycin across the period from 2017 to 2025 were significantly different (*p* < 0.001, Table 3). The resistance to the two drugs exhibited similar annual fluctuation patterns: both agents exhibited a high resistance in 2019 and 2025 and a low resistance in 2021 and 2022. For erythromycin, MICs in 2019 were significantly higher than those in 2018 (*p* = 0.035), 2022 (*p* = 0.048), and 2024 (*p* = 0.009); MICs in 2025 were also significantly higher than those in 2024 (*p* = 0.021, Figure 1A). For azithromycin, MIC values in 2019 and 2025 were significantly higher than those in 2021 (*p* = 0.039 and *p* = 0.018, respectively; Figure 1B). Moreover, MICs in 2023 were significantly higher than those in 2021 (*p* < 0.001) and 2022 (*p* = 0.005).

An analysis based on a stacked percentage bar chart (Figure 1A) revealed that the proportion of high-level erythromycin-resistant *M. pneumoniae* isolates fluctuated in a “rise–fall–rise” pattern during 2017–2025. In 2019 and 2025, the proportion of high-level resistant isolates exceeded 89%, with ultra-high-resistance isolates (1024 μg/mL) predominating. No 1024 μg/mL isolates were detected in 2022, and the proportion of high-level resistant isolates decreased to 66.7% (12/18) before dropping further to 51.9% (14/27) in 2024. Low-level resistant isolates were sporadically detected in only 3 years, accounting for less than 7% each year.

For the distribution characteristics of azithromycin MIC values (Figure 1B), low-level resistant isolates persisted consistently. Moderate-level resistant isolates were predominant in 2017, 2018, 2023, and 2024. In 2019 and 2025, the isolates exhibited significant resistance differentiation with relatively balanced proportions between different levels. In 2021, low-level resistant isolates accounted for the highest proportion (61.5%, 8/13), with no high-level resistant isolates detected. Notably, the proportion of high-level azithromycin-resistant isolates peaked in 2025 (31.3%, 5/16). Collectively, the azithromycin resistance level displayed obvious annual fluctuations and was significantly lower than that of erythromycin.

Overall, *M. pneumoniae* remained susceptible to tetracycline, levofloxacin and moxifloxacin from 2017 to 2025. MIC values fluctuated, with statistically significant differences observed across years. For tetracycline and moxifloxacin, MIC_90_ was highest in 2019 (Table 3). Tetracycline MICs in 2019 were significantly higher than those in 2018 (*p* = 0.014), 2021 (*p* = 0.019) and 2022 (*p* = 0.009). Moxifloxacin MICs in 2019 were significantly higher than those in 2017 (*p* < 0.001), 2018 (*p* < 0.001), 2021 (*p* = 0.014), 2022 (*p* = 0.003), 2023 (*p* < 0.001), and 2024 (*p* < 0.001). For levofloxacin, the narrowest and lowest MIC range (≤0.125–0.5 μg/mL) was observed in 2025, and MICs in 2025 were significantly lower than those in 2017 (*p* = 0.02), 2023 (*p* = 0.028) and 2024 (*p* = 0.006) (Table 3).

### 2.3. Comparison of Antimicrobial Susceptibility in Macrolide-Resistant Mycoplasma pneumoniae Isolates Across Different Epidemic Phases

The analysis of the MIC values of erythromycin and azithromycin against MRMP isolates from different epidemic phases showed that erythromycin exhibited high-level resistance in all phases. During the low epidemic phase (2017–2018) and epidemic outbreak phase (2023–2024), the MIC_50_ and MIC_90_ of erythromycin were 512 μg/mL and 1024 μg/mL, respectively (Table 4). Both the MIC_50_ and MIC_90_ of erythromycin reached 1024 μg/mL in the epidemic initiation phase (2019–2020) and epidemic recovery phase (2025), while both values were 512 μg/mL in the ultra-low epidemic phase (2021–2022) (Table 4). The overall azithromycin resistance level was lower than that of erythromycin: the MIC_50_ was 64 μg/mL in the low epidemic, epidemic initiation, and epidemic outbreak phases, which decreased to 16 μg/mL in the ultra-low epidemic phase (Table 4). Notably, the MIC_50_ and MIC_90_ of azithromycin surged to 128 μg/mL and 512 μg/mL in 2025, indicating high-level resistance (Table 4).

The analysis of the distribution characteristics of erythromycin and azithromycin MIC values across different epidemic phases revealed that statistical differences existed (*p* < 0.001). Similarly, the proportion of ultra-high-level erythromycin-resistant and high-level azithromycin-resistant *M. pneumoniae* isolates fluctuated in a “rise–fall–rise” pattern.

**Figure 2:**
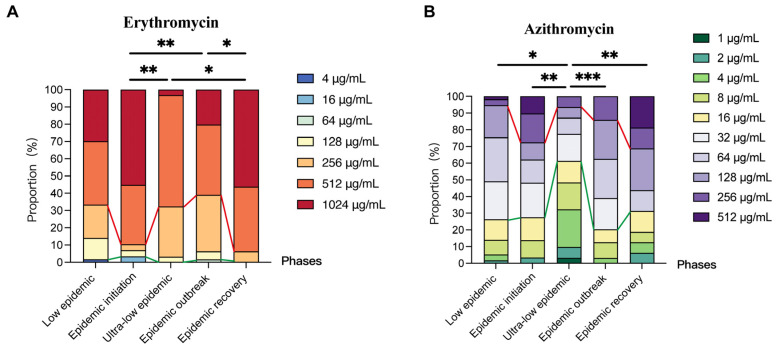
Distribution characteristics of erythromycin and azithromycin MICs in *Mycoplasma pneumoniae* isolates across different phases in Beijing, China. Low epidemic phase: 2017–2018; Epidemic initiation phase: 2019–2020; Ultra-low epidemic phase: 2021–2022; Epidemic outbreak phase: 2023–2024; Epidemic recovery phase: 2025. Strains below the green line were low-concentration resistant, and those above the red line were high-concentration resistant. (**A**) Erythromycin; (**B**) Azithromycin. * *p* < 0.05, ** *p* < 0.01, *** *p* < 0.001.

For erythromycin, MICs were significantly higher in the epidemic initiation and epidemic recovery phases than in the ultra-low epidemic and epidemic outbreak phases (epidemic initiation vs. ultra-low epidemic: (*p* = 0.005); epidemic initiation vs. outbreak: (*p* = 0.005); recovery vs. ultra-low epidemic: (*p* = 0.011); and recovery vs. outbreak: (*p* = 0.016)) (Figure 2A). During initiation and recovery, the proportion of medium-level resistant strains decreased, while high-level resistant strains increased dramatically to over 85%, particularly ultra-high resistant strains (MIC = 1024 μg/mL).

For azithromycin, MICs were significantly lower in the ultra-low epidemic phase than in all other phases (vs. low epidemic: (*p* = 0.03); vs. epidemic initiation: (*p* = 0.006); vs. epidemic outbreak: (*p* < 0.001); vs. epidemic recovery: (*p* = 0.004)) (Figure 2B). In the epidemic initiation phase and epidemic recovery phase, the proportions of isolates with three resistance levels were relatively balanced. In the low epidemic phase and outbreak phase, moderate-level resistant isolates were predominant. Particularly, high-level resistant isolates rose from 5.26% (3/57) in the low epidemic phase to 27.6% (8/29) at epidemic initiation, declined to 6.45% (2/31) in the ultra-low epidemic phase, and then exhibited an increasing trend, reaching 14.1% (9/64) in the outbreak phase and 31.25% (5/16) in the recovery phase.

Statistically significant differences in MIC values were observed for tetracycline and quinolones across different phases. For tetracycline, MIC_50_ (0.5 μg/mL) and MIC_90_ (1 μg/mL) were highest in the epidemic initiation phase (Table 4), with MICs significantly higher than those in ultra-low (*p* < 0.001) and low epidemic phases (*p* = 0.006). For moxifloxacin, MIC_90_ (0.5 μg/mL) was also highest in the epidemic initiation phase (Table 4), with MICs significantly higher than those in ultra-low (*p* < 0.001), low epidemic (*p* < 0.001) and outbreak phases (*p* < 0.001). For levofloxacin, MICs in the recovery phase were significantly lower than those in low epidemic (*p* = 0.016) and outbreak phases (*p* = 0.001). In addition, MICs in the initiation phase were significantly lower than those in the outbreak phase (*p* = 0.019).

### 2.4. Comparison of Antimicrobial Susceptibility in Macrolide-Resistant Mycoplasma pneumoniae Isolates Across Different Age Groups

Across all three age groups, the erythromycin MIC ranges of strains were 128–1024 μg/mL, 128–1024 μg/mL, and 4–1024 μg/mL, respectively. The MIC_50_ and MIC_90_ values were uniformly 512 μg/mL and 1024 μg/mL (Table 5). The overall proportion of low-level strains was relatively low.

**Figure 3:**
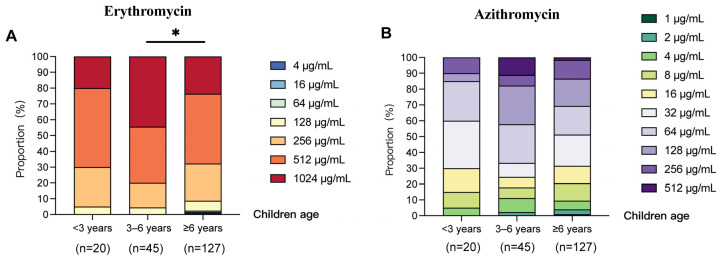
Distribution characteristics of erythromycin and azithromycin MICs in *Mycoplasma pneumoniae* isolates from children across different age groups in Beijing, China, 2017–2025. (**A**) Erythromycin; (**B**) Azithromycin. * *p* < 0.05.

For erythromycin, MIC distributions differed significantly between age groups (*p* = 0.036). The proportion of high-level resistant strains was highest in the 3–6 year group, and MICs in this group were also significantly higher than those in the ≥6 year group (*p* = 0.034). Medium-level resistant strains were the least prevalent in the 3–6 year group (20.0%, 9/45), though the proportions were higher at approximately 30.0% in the <3 year and ≥6 year groups (6/20 and 38/127, respectively). Low-level resistant strains were only detected in the ≥6 year group, accounting for 2.36% (3/127) (Figure 3A).

The azithromycin MIC ranges of MRMP strains isolated from the above age groups were 4–256 μg/mL, 2–512 μg/mL, and 1–512 μg/mL, with MIC_50_ values of 32 μg/mL, 64 μg/mL, and 32 μg/mL and MIC_90_ values of 128 μg/mL, 512 μg/mL, and 256 μg/mL, respectively (Table 4). Although statistically significant differences between age groups were not found, the distribution patterns were similar to those of erythromycin. The proportion of high-level resistant strains was highest in the 3–6 year group (17.7%, 8/45). The proportions of medium-level resistant strains were similar within three age groups, at 60% (12/20), 57.8% (26/45), and 55.1% (70/127), respectively. The proportion of low-level resistant strains was highest in the ≥6 year group (31.5%, 40/127).

The MIC range of tetracycline varied among age groups, whereas those of levofloxacin and moxifloxacin remained unchanged. MIC_50_ and MIC_90_ of these three antibiotics were consistent across age groups, with no significant differences found.

These results suggested that children aged 3–6 years constituted the primary population with a high-level resistance to erythromycin and azithromycin.

## 3. Discussion

Non-pharmacological interventions during the COVID-19 pandemic, such as mask use, social distancing, school closures, and improved ventilation, effectively reduced respiratory transmission and resulted in a significant decrease in the detection rate of *M. pneumoniae*. However, this rate has rebounded since April 2023, with large-scale outbreaks emerging in China, Denmark, the Netherlands, the United States, and other countries in October 2023—at which point the detection rate of MP in children with respiratory tract infections in Beijing increased, reaching levels as high as 61.1% among hospitalized children [9,10,11,12,13,16]. Macrolide antibiotics act on domain V of the 23S rRNA within the bacterial 50S ribosomal subunit and block bacterial protein synthesis by promoting the premature dissociation of peptidyl-tRNA from the ribosome [17]. Given the unique physiological characteristics of children in their developmental stage, this class of antibiotics is designated as the first-line therapeutic agent for MP infections in children. *M. pneumoniae* resistance is currently considered to be closely associated with target site alterations, active drug efflux, and drug inactivation [18].

In this study, we found that 100% of the *M. pneumoniae* strains harbored the A2063G mutation in the 23S rRNA gene, which further confirms that MRMP strains predominate in *M. pneumoniae* epidemics in China. Overuse and inappropriate use of macrolides in viral respiratory infections may have increased antimicrobial selection pressure, thereby promoting the emergence and spread of drug-resistant *M. pneumoniae* strains. Numerous studies have demonstrated a strong association between this mutation and high-level resistance to macrolide antibiotics [19,20]. Zhao et al. reported that 53 *M. pneumoniae* isolates from Beijing during 2014–2016 were resistant to erythromycin and azithromycin, with MIC ranges of ≥256 μg/mL and 2–64 μg/mL, respectively [21]. In the present study, the resistance levels of *M. pneumoniae* to erythromycin and azithromycin during 2017–2025 were higher than those observed in the 2014–2016 period. Furthermore, the MIC_50_ of erythromycin for the strains was consistently ≥512 μg/mL, and the MIC_90_ reached 1024 μg/mL in most years, indicating that the strains exhibit high-level erythromycin resistance with an extremely high risk of clinical treatment failure. For 197 clinical isolates, the azithromycin MIC range was 2–512 μg/mL (MIC_50_ = 64 μg/mL), with a significantly lower resistance level compared to erythromycin; the MIC_50_ and MIC_90_ of erythromycin were 8-fold and 4-fold higher than those of azithromycin, respectively. The differential resistance of MRMP to erythromycin and azithromycin may be associated with differences in the clinical use frequency of the two drugs and the selective pressure on drug-resistant mutation sites.

Previous studies have shown that during 2014–2018, the MIC ranges of tetracycline and levofloxacin against *M. pneumoniae* strains in China were 0.016–0.5 μg/mL and 0.125–1 μg/mL, respectively [21,22]. During 2017–2019, the MIC ranges of tetracycline, levofloxacin, and moxifloxacin against MP isolates from Shanghai were 0.06–2 μg/mL, 0.03–1 μg/mL, and 0.015–0.25 μg/mL, with corresponding MIC_50_/MIC_90_ values of 0.5/1 μg/mL, 0.5/1 μg/mL, and 0.125/0.25 μg/mL [23]. All *M. pneumoniae* strains in this study remained highly susceptible to tetracycline, levofloxacin, and moxifloxacin, which is consistent with previous findings. Tetracycline antibiotics (doxycycline and minocycline) and quinolone antibiotics (levofloxacin and moxifloxacin) serve as the main alternative therapeutic agents for MPP, particularly for refractory MPP, macrolide-unresponsive MPP, and severe MPP. Considering that these two classes of drugs may induce adverse reactions, such as dental discoloration and impaired enamel development, off-label use requires the use of informed consent forms [24]. Significantly higher MICs of tetracycline and moxifloxacin were observed in 2019, which fell in the epidemic initiation phase. Levofloxacin MICs were significantly higher in 2023–2024 (outbreak phase) compared to 2025. This may be related to the increased detection rate of macrolide-resistant *M. pneumoniae* during epidemic periods, leading physicians to select alternative antibiotics for empirical therapy.

For 34 MRMP isolates from Beijing during 2017–2018, Zhao et al. reported that the MIC ranges of erythromycin and azithromycin were 128–>256 μg/mL and 2–32 μg/mL, respectively [22]. Wang et al. found that 97.3% of 182 MP isolates from Shanghai during 2017–2019 were resistant to erythromycin (MIC ≥ 64 μg/mL), with the macrolide resistance rate increasing from 85.7% in 2017 to 100% in 2019 [23]. Jiang et al. investigated the resistance of 45 MRMP isolates during 2015–2019, reporting that the MIC ranges of erythromycin and azithromycin were 64–1024 μg/mL and 128–1024 μg/mL in 2017–2018 and 512–1024 μg/mL and 128–512 μg/mL in 2019, respectively [25]. The results of the present study demonstrated that MP strains exhibit high-level erythromycin resistance, and the proportion of different levels of resistant strains is highly positively correlated with the epidemic intensity, which is consistent with the findings of Jiang et al. For both erythromycin and azithromycin, the epidemic initiation phase and epidemic recovery phase both exhibited a decrease in medium-level resistant strains accompanied by an increase in highly resistant strains. The proportion of ultra-high erythromycin-resistant strains and highly azithromycin-resistant strains exhibited a “rise–fall–rise” trend during 2017–2025. In the ultra-low epidemic phase, the proportion of ultra-high erythromycin-resistant strains (MIC = 1024 μg/mL) was the lowest, and the proportion of low-level resistant azithromycin was highest. These phenomena indicated that clustered infections in children intensified the transmission of resistant strains, and the reduction in strain transmission can alleviate the selective pressure of antibiotics and delay the evolution of drug resistance. In addition, strengthening infection control in places where children gather and reducing strain transmission during epidemic periods are key non-pharmaceutical interventions that can be used to delay the evolution of macrolide resistance. Interestingly, in the epidemic outbreak phase, the proportion of ultra-high resistant strains increased, but the proportion of high-level resistant strains slightly decreased compared with the ultra-low epidemic phase. This may be attributed to the evolution of physicians’ prescription patterns in 2024, which collectively resulted from guideline updates, widespread availability of MRMP detection, empirical prescribing habits, and dynamic changes in local epidemic and resistance patterns, thereby leading to a reduction in the detection of high-level resistant strains that year. Although erythromycin and azithromycin exhibited similar trends over time, the clinical use of erythromycin requires careful evaluation due to its consistently high MIC values. Azithromycin can be used as a first-line agent during the ultra-low and low prevalence periods but should be administered in conjunction with MIC testing; its resistance level rises substantially in the recovery period, and alternative drugs, such as tetracyclines and moxifloxacin, can be used in accordance with expert consensus (with strict adherence to age-related medication restrictions for children) [24]. The low resistance rates and high susceptibility of *M. pneumoniae* to fluoroquinolones and tetracyclines observed in this study are most likely attributed to their limited clinical use in pediatrics. Tetracyclines are contraindicated in young children due to the risk of tooth discoloration and impaired bone development, while fluoroquinolones are generally avoided because of potential articular cartilage toxicity. The restricted use of these antibiotics has reduced antimicrobial selection pressure, thus maintaining low resistance levels.

This study also identified significant differences in the erythromycin resistance of MRMP among different age groups, which provides an important basis for individualized medication in children. For erythromycin, MIC distributions differed significantly across age groups. The proportion of high-level resistant strains was highest in the 3–6 year group, and MICs in this group were also significantly higher than those in the ≥6 year group (*p* = 0.034). Although a statistically significant difference was not identified between the different age groups for azithromycin, the 3–6 year group had the highest proportion of high-level resistant strains and the highest MIC_50_ and MIC_90_, suggesting that this group may be facing a more serious azithromycin resistance problem. This may be closely associated with frequent clustered infections and empirical use in the 3–6 year group, where sustained selective pressure accelerates the transmission and expansion of resistant strains. And in the ≥6 year group, the proportion of low-level resistant strains was the highest, both for erythromycin and azithromycin, which may be due to the greater emphasis on antibiotic susceptibility-guided medication in this age group and the increased use of quinolones and tetracyclines, thereby alleviating the selective pressure of macrolides on MP. Collectively, our results showed that clinical practice should implement precision medication based on age characteristics and MIC testing results.

This study has some limitations that should be noted. First, it is a single-center study with all *M. pneumoniae* strains isolated from pediatric patients in Beijing, lacking multi-center data from different regions. This makes it difficult to fully reflect the epidemiological characteristics of pediatric *M. pneumoniae* drug resistance regionally. Second, no systematic clinical baseline subject data (e.g., antimicrobial history, underlying diseases, treatment regimens and prognosis) were collected, which precludes an analysis of the correlation between clinical drug selection pressure and drug resistance and the evaluation of drug resistance’s impact on clinical treatment outcomes. Third, all isolates included in this study were macrolide-resistant, and no susceptible strains were detected. This may represent a selection bias due to the high prevalence of resistant strains and long-term antimicrobial pressure in the region.

## 4. Materials and Methods

### 4.1. Isolation of M. pneumoniae Strains

A total of 197 *M. pneumoniae* strains were isolated from respiratory specimens of children with *M. pneumoniae* pneumonia (MPP) in 2017–2025. The specimens were collected from outpatients and inpatients at the Capital Center for Children’s Health, Capital Medical University, Capital Institute of Pediatrics, including bronchoalveolar lavage fluid (BALF), pharyngeal swabs, and sputum. The period 2017–2025 (nine years) covered a complete cycle of predominant genotype shift from P1-1 to P1-2 and back to P1-1, which is highly relevant for analyzing the dynamic changes in drug resistance. According to the long-term continuous surveillance data from our laboratory over the past 40 years combined with previously published epidemiological evidence, we divided 2017–2025 into five phases: the low epidemic phase (2017–2018), epidemic initiation phase (2019–2020), ultra-low epidemic phase (COVID-19 control period, 2021–2022), epidemic outbreak phase (2023–2024), and epidemic recovery phase (2025) [6,7,8,9,10,11,12,13]. Patients were divided into three age groups: infants aged <3 years, preschool children aged 3–6 years, and school-age children aged ≥6 years.

### 4.2. Detection of M. pneumoniae and Macrolide Resistance Mutations

*Mycoplasma pneumoniae* nucleic acid and macrolide resistance-associated point mutations (A2063G/A2064G) in the specimens were detected using *Mycoplasma pneumoniae* and the Macrolide-Resistant Isolate Diagnostic Kit (PCR Fluorescence Probing; Mole BioScience Co., Ltd., Jiangsu, China), in accordance with the manufacturer’s instructions. The 25 μL reaction system was composed of 6.0 μL of buffer, 2.0 μL of primer–probe mix, 0.5 μL of enzyme, 11.5 μL of double-distilled water (DDW), and 5 μL of template. The PCR reaction protocol was as follows: initial incubation at 50 °C for 2 min and 95 °C for 2 min followed by 40 cycles of denaturation at 91 °C for 15 s and annealing/extension at 64 °C for 1 min. The test results were determined in accordance with the manufacturer’s instructions.

### 4.3. Isolation and Culture of M. pneumoniae

MP-positive respiratory specimens were mixed with PPLO liquid medium (Becton, Dickinson and Company, Sparks, MD, USA) at a volume ratio of 1:10 in sterile glass test tubes and incubated at 37 °C. Blind passage was performed once every 7 days. *M. pneumoniae* growth was indicated when the phenol red-containing liquid medium changed color from red to orange-yellow and remained clear.

### 4.4. Antimicrobial Susceptibility Testing of M. pneumoniae

The minimum inhibitory concentrations (MICs) of five antimicrobial agents (erythromycin, azithromycin, tetracycline, levofloxacin, and moxifloxacin) were determined using the broth microdilution method, in accordance with the guidelines recommended by the Clinical and Laboratory Standards Institute (CLSI) 2011 (CLSI M43–A) [26]. Standard reference substances of the above five antimicrobial agents were dissolved and subjected to serial two-fold dilutions to prepare a series of concentration gradients. *M. pneumoniae* bacterial suspensions (containing 10^5^ colony-forming units per mL) were mixed with the antimicrobial agents at different concentrations and incubated at 37 °C. The lowest concentration of each agent that completely inhibited *M. pneumoniae* growth was recorded as the MIC value. The susceptibility breakpoints were defined as follows: erythromycin (MIC ≤ 0.5 μg/mL), azithromycin (MIC ≤ 0.5 μg/mL), tetracycline (MIC ≤ 2 μg/mL), levofloxacin (MIC ≤ 1 μg/mL), and moxifloxacin (MIC ≤ 0.5 μg/mL). The MP reference strain M129 (ATCC 29342) was used as the quality control strain. Meanwhile, a drug-free blank control and a sterile fluid negative control were set up for each assay.

### 4.5. Statistical Analysis

All data were statistically analyzed using SPSS (version 30.0) software. The MIC range, MIC_50_, and MIC_90_ of the strains were calculated, and categorical data were expressed as percentages. Kruskal–Wallis or Mann–Whitney U tests were used to compare the distribution of MICs across different years, pediatric age groups, and epidemic phases. A *p* value < 0.05 was considered statistically significant.

## 5. Conclusions

This study clarifies the associations between the macrolide resistance of pediatric MRMP strains and epidemic intensity as well as age, providing critical data supporting clinical precision medication and the prevention and control of drug resistance. The resistance of *M. pneumoniae* to macrolides in children is closely associated with the epidemic intensity and age. Erythromycin is no longer suitable as an empirical therapy for MP infections during epidemic periods, whereas azithromycin can be cautiously administered in young children according to age stratification and MIC detection results. Furthermore, our study covers a complete predominant genotype shift cycle (2017–2025, nine years) from P1-1 to P1-2 and back to P1-1. Meanwhile, it is imperative that we strengthen the prevention and control of cluster MP infections during epidemic phases to reduce the transmission of drug-resistant MP strains.

## Figures and Tables

**Table 1:** Antimicrobial Susceptibility of *Mycoplasma pneumoniae* isolates from children in Beijing, China, 2017–2025.

Agents	No. of Resistant Isolates	Resistance Rate	MIC Range (μg/mL)	MIC_50_(μg/mL)	MIC_90_ (μg/mL)	MIC of M129 (μg/mL)
Erythromycin	197	100%	4–1024	512	1024	0.0015625
Azithromycin	197	100%	1–512	64	256	0.03125
Tetracycline	0	0.00%	≤0.125–2	0.5	0.5	0.0625
Levofloxacin	0	0.00%	≤0.125–1	0.5	1	0.0625
Moxifloxacin	0	0.00%	≤0.125–0.5	≤0.125	≤0.125	0.03125

**Table 2:** MICs distribution of macrolide drugs in *Mycoplasma pneumoniae* isolates from children in Beijing, China, 2017–2025.

Macrolides	MIC [μg/mL, No. (%)]											Total
	1	2	4	8	16	32	64	128	256	512	1024	
Erythromycin	0 (0%)	0 (0%)	1 (0.51%)	0 (0%)	1 (0.51%)	0 (0%)	1 (0.51%)	12 (6.09%)	43 (21.8%)	83 (42.1%)	56 (28.4%)	197
Azithromycin	1 (0.51%)	5 (2.53%)	12 (6.09%)	20 (10.2%)	22 (11.2%)	36 (18.3%)	39 (19.8%)	35 (17.8%)	20 (10.2%)	7 (3.55%)	0 (0%)	197

**Table 3:** MICs of different antibiotics in *Mycoplasma pneumoniae* isolates from children in Beijing across different years.

Macrolides	Year	No. of Isolates	MIC Range (μg/mL)	MIC_50_ (μg/mL)	MIC_90_ (μg/mL)	*p* Value
Erythromycin	2017	31	128–1024	512	1024	<0.001
	2018	26	4–1024	512	1024	
	2019	29	16–1024	1024	1024	
	2021	13	128–1024	512	512	
	2022	18	256–512	512	512	
	2023	37	64–1024	512	1024	
	2024	27	128–1024	512	1024	
	2025	16	256–1024	1024	1024	
Azithromycin	2017	31	4–256	64	128	<0.001
	2018	26	2–512	32	128	
	2019	29	2–512	64	512	
	2021	13	1–128	8	64	
	2022	18	2–256	16	256	
	2023	37	4–256	128	256	
	2024	27	8–256	32	128	
	2025	16	2–512	128	512	
Tetracycline	2017	31	≤0.125–0.5	0.25	0.5	0.001
	2018	26	≤0.125–1	0.25	0.5	
	2019	29	≤0.125–2	0.5	1	
	2021	13	≤0.125–0.5	0.25	0.25	
	2022	18	≤0.125–0.5	≤0.125	0.5	
	2023	37	≤0.125–1	0.25	0.5	
	2024	27	≤0.125–0.5	0.25	0.5	
	2025	16	≤0.125–1	0.25	0.5	
Levofloxacin	2017	31	0.25–1	0.5	1	<0.001
	2018	26	0.25–1	0.5	0.5	
	2019	29	≤0.125–1	0.5	1	
	2021	13	≤0.125–1	0.5	1	
	2022	18	0.25–1	0.5	0.5	
	2023	37	0.25–1	0.5	1	
	2024	27	0.5–1	0.5	1	
	2025	16	≤0.125–0.5	0.5	0.5	
Moxifloxacin	2017	31	≤0.125	≤0.125	≤0.125	<0.001
	2018	26	≤0.125	≤0.125	≤0.125	
	2019	29	≤0.125–0.5	≤0.125	0.5	
	2021	13	≤0.125	≤0.125	≤0.125	
	2022	18	≤0.125	≤0.125	≤0.125	
	2023	37	≤0.125	≤0.125	≤0.125	
	2024	27	≤0.125	≤0.125	≤0.125	
	2025	16	≤0.125–0.25	≤0.125	≤0.125	

**Table 4:** MICs of different antibiotics in *Mycoplasma pneumoniae* isolates from children in Beijing across different phases.

Macrolides	Epidemic Phases	No. of Isolates	MIC Range (μg/mL)	MIC_50_ (μg/mL)	MIC_90_ (μg/mL)	*p* Value
Erythromycin	Low epidemic phase (2017–2018)	57	4–1024	512	1024	<0.001
	Epidemic initiation phase (2019–2020)	29	16–1024	1024	1024	
	Ultra-low epidemic phase (2021–2022)	31	128–1024	512	512	
	outbreak phase (2023–2024)	64	64–1024	512	1024	
	epidemic recovery phase (2025)	16	256–1024	1024	1024	
Azithromycin	Low epidemic phase (2017–2018)	57	2–256	64	128	<0.001
	Epidemic initiation phase (2019–2020)	29	2–512	64	512	
	Ultra-low epidemic phase (2021–2022)	31	1–256	16	128	
	outbreak phase (2023–2024)	64	4–256	64	256	
	epidemic recovery phase (2025)	16	2–512	128	512	
Tetracycline	Low epidemic phase (2017–2018)	57	≤0.125–1	0.25	0.5	<0.001
	Epidemic initiation phase (2019–2020)	29	≤0.125–2	0.5	1	
	Ultra-low epidemic phase (2021–2022)	31	≤0.125–0.5	0.25	0.5	
	outbreak phase (2023–2024)	64	≤0.125–1	0.25	0.5	
	epidemic recovery phase (2025)	16	≤0.125–1	0.25	0.5	
Levofloxacin	Low epidemic phase (2017–2018)	57	0.25–1	0.5	1	<0.001
	Epidemic initiation phase (2019–2020)	29	≤0.125–1	0.5	1	
	Ultra-low epidemic phase (2021–2022)	31	≤0.125–1	0.5	1	
	outbreak phase (2023–2024)	64	0.25–1	0.5	1	
	epidemic recovery phase (2025)	16	≤0.125–0.5	0.5	1	
Moxifloxacin	Low epidemic phase (2017–2018)	57	≤0.125	≤0.125	≤0.125	<0.001
	Epidemic initiation phase (2019–2020)	29	≤0.125–0.5	≤0.125	0.5	
	Ultra-low epidemic phase (2021–2022)	31	≤0.125	≤0.125	≤0.125	
	outbreak phase (2023–2024)	64	≤0.125	≤0.125	≤0.125	
	epidemic recovery phase (2025)	16	≤0.125–0.5	≤0.125	≤0.125	

**Table 5:** MICs of different antibiotics in *Mycoplasma pneumoniae* isolates from children across different age groups in Beijing, China (2017–2025).

Macrolides	Age Groups	No. of Isolates	MIC Range (μg/mL)	MIC_50_ (μg/mL)	MIC_90_ (μg/mL)	*p* Value
Erythromycin	<3 years	20	128–1024	512	1024	0.036
	3–6 years	45	128–1024	512	1024	
	≥6 years	127	4–1024	512	1024	
Azithromycin	<3 years	20	4–256	32	128	0.173
	3–6 years	45	2–512	64	512	
	≥6 years	127	1–512	32	256	
Tetracycline	<3 years	20	≤0.125–1	0.25	0.5	0.336
	3–6 years	45	≤0.125–2	0.25	0.5	
	≥6 years	127	≤0.125–2	0.25	0.5	
Levofloxacin	<3 years	20	≤0.125–1	0.5	1	0.879
	3–6 years	45	≤0.125–1	0.5	1	
	≥6 years	127	≤0.125–1	0.5	1	
Moxifloxacin	<3 years	20	≤0.125–0.5	≤0.125	≤0.125	0.995
	3–6 years	45	≤0.125–0.5	≤0.125	≤0.125	
	≥6 years	127	≤0.125–0.5	≤0.125	≤0.125	

## Data Availability

The original contributions presented in this study are included in the article. Further inquiries can be directed to the corresponding author(s).

## Abbreviations

The following abbreviations are used in this manuscript:

*M. pneumoniae*	*Mycoplasma pneumoniae*
MP	*Mycoplasma pneumoniae*
MRMP	macrolide-resistant *Mycoplasma pneumoniae*
MPP	*Mycoplasma pneumoniae* pneumonia
MIC	minimum inhibitory concentration
